# Supplementary material for: Apoε4 disrupts neurovascular regulation and undermines white matter integrity and cognitive function
Source: Nat Commun. 2018 Sep 19;9:3816. doi: 10.1038/s41467-018-06301-2 (PMC6145902; doi:10.1038/s41467-018-06301-2)
Supplement: Supplementary file 1 — Supplementary Information [file 41467_2018_6301_MOESM1_ESM.pdf]

Supplementary Information

Apoε4 distrupts neurovascular regulation and undermines white matter integrity and cognitive function.

Koizumi and Hattori et al.

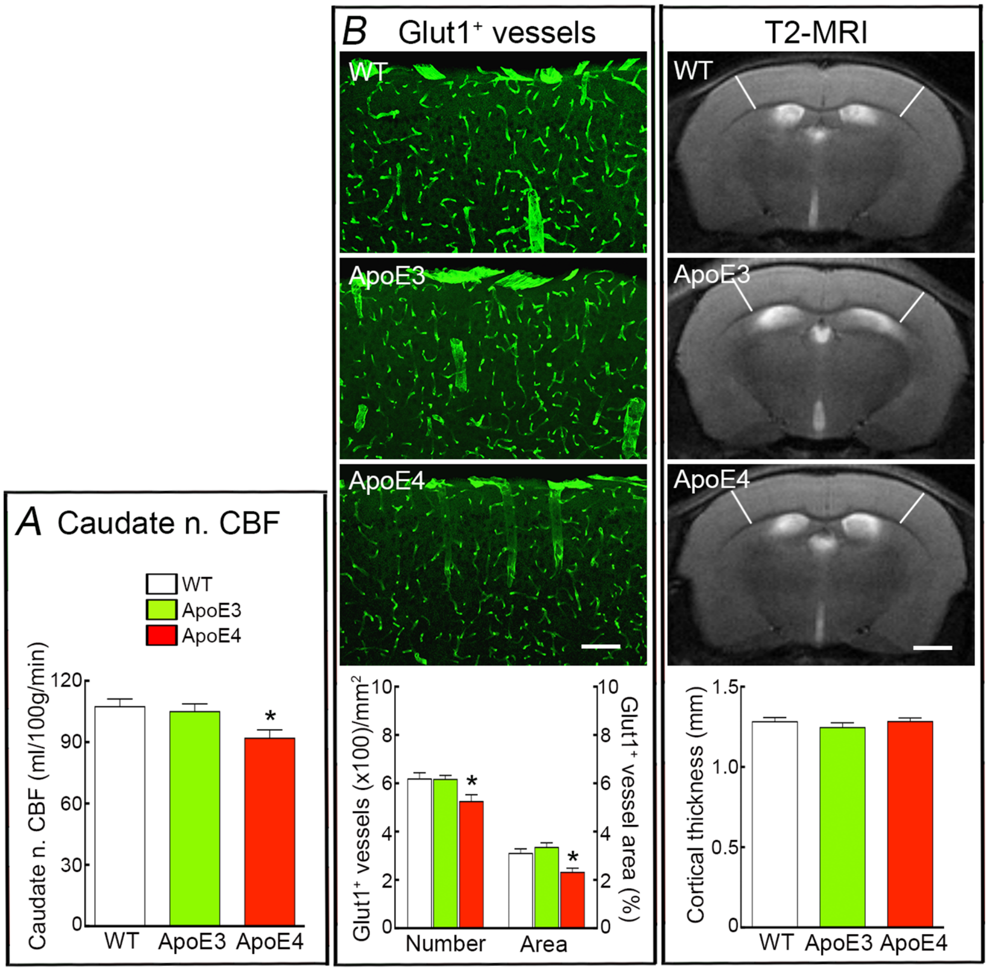

Supplementary Figure 1. ApoE4-TR mice show reduced resting CBF in caudate nucleus and reduced vascular density in the neocortex. ***A***. Caudate nucleus CBF, assessed by ASL-MRI, is lower in ApoE4-TR mice, compared to WT and ApoE3-TR mice. ***B***. Cortical Glut-1^{+} microvessels are reduced in ApoE4-TR mice, compared to WT and ApoE3-TR mice. Scale bar=100 μm. ***C***. Cortical thickness, assessed bilaterally in MRI scans at the level of the somatosensory cortex (-0.7 to 0.38 from bregma), is comparable in ApoE4-TR, ApoE3-TR, and WT mice. Scale bar=1 mm; N=5/group in ***A*** and ***B***; N=10/group in ***C***; *$p<0.05$, one-way ANOVA and Tukey's test.

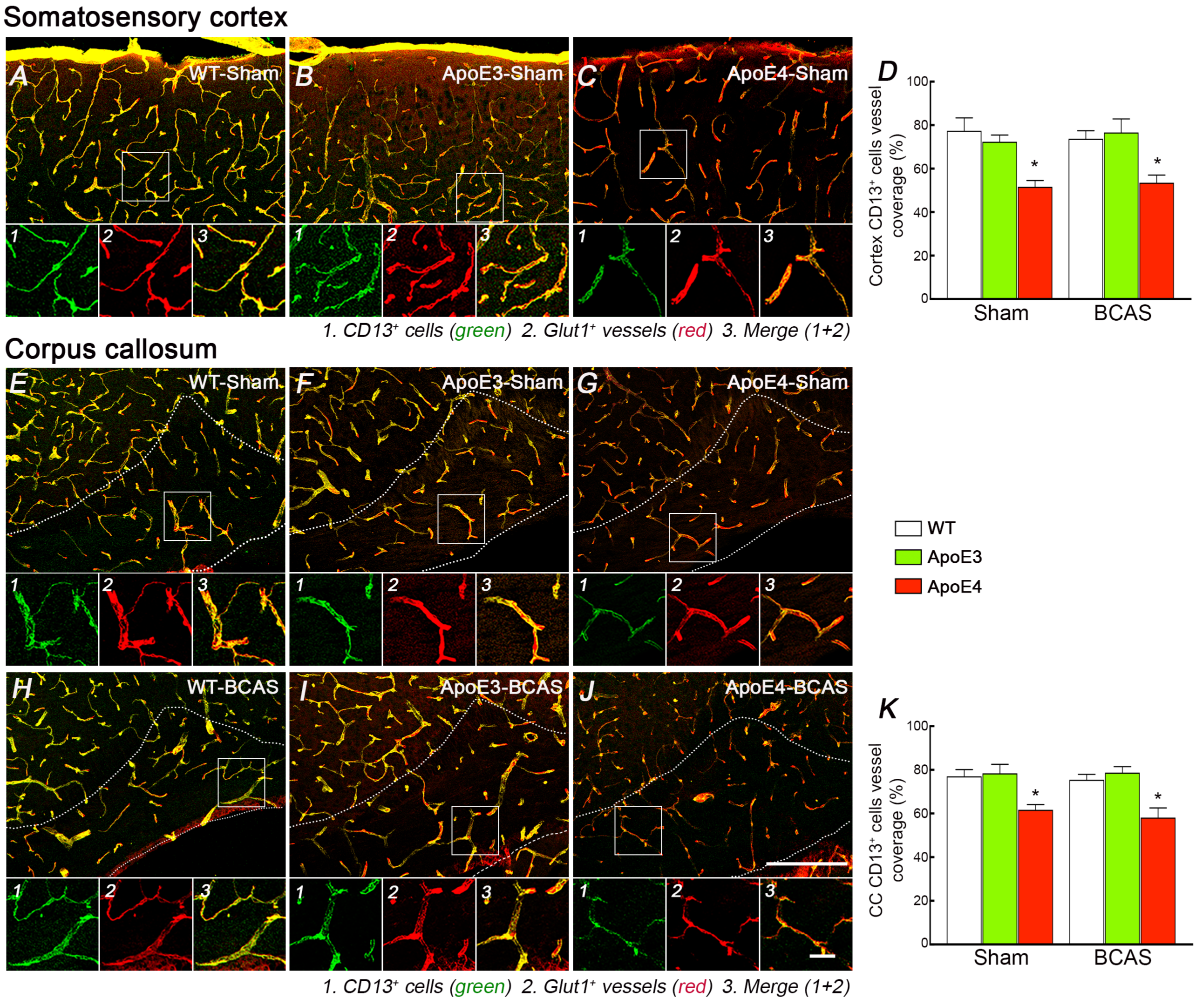

Supplementary Figure 2. ApoE4-TR mice show reduced vascular coverage of CD13+ mural cells (pericytes) in somatosensory cortex and CC. Representative merged images of CD13+ vascular mural cells and Glut-1+ blood vessels (***A-C*** and ***E-J***) in somatosensory cortex (***A-C***) and CC (***E-J***) after sham surgery or BCAS. Inserts at the bottom of each panel represent enlagements of the area outlined by the box immunolabeled with CD13 (1), Glut1 (2) or merged (3). Quantification of the vascular area covered by CD13+ cells (expressed as %) is presented in panels ***D*** and ***K***. ApoE4-TR mice show reduced coverage of CD13+ vascular mural cells after sham surgery, but no further reduction after BCAS. Data represent mean±sem. Scale bars in J and in 3 are 250 µm and 50 µm, respectively. *$p<0.05$ from WT and ApoE3; N=5/group; one way ANOVA and Tukey's test.

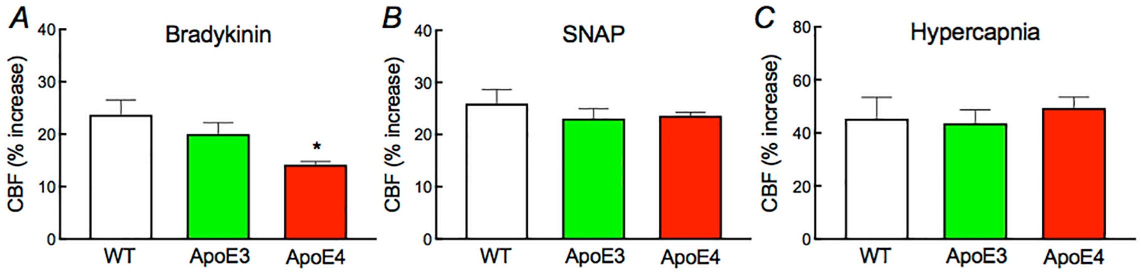

Supplementary Figure 3. ApoE4-TR mice show significant impairment of neurovascular function. ApoE4-TR mice show significant impairment in the increase in CBF induced by neocortical application of the endothelium-dependent vasodilator bradykinin (***A***), but not in the CBF response to the NO donor SNAP (***B***), or by hypercapnia (***C***). N=5/group; * $p<0.05$ from WT or ApoE3; one-way ANOVA and Tukey's test.

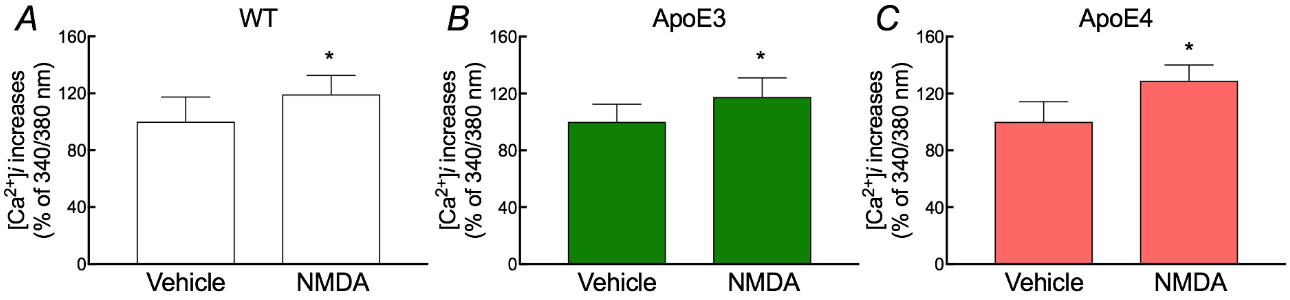

Supplementary Figure 4. NMDA-induced increase of intracellular Ca^{2+} in isolated cortical neurons is not reduced in ApoE4-TR mice. Increases in intracellular Ca^{2+} $[Ca^{2+}]i$ elicited by NMDA (40 μM) in isolated cortical neurons is comparable among WT (***A***), ApoE3-TR (***B***), and ApoE4-TR (***C***) mice. Data are shown as mean±sem (N=10 neurons/group from 3 independent isolations). *$p<0.05$ from vehicle in each group; paired two-tailed T-test.

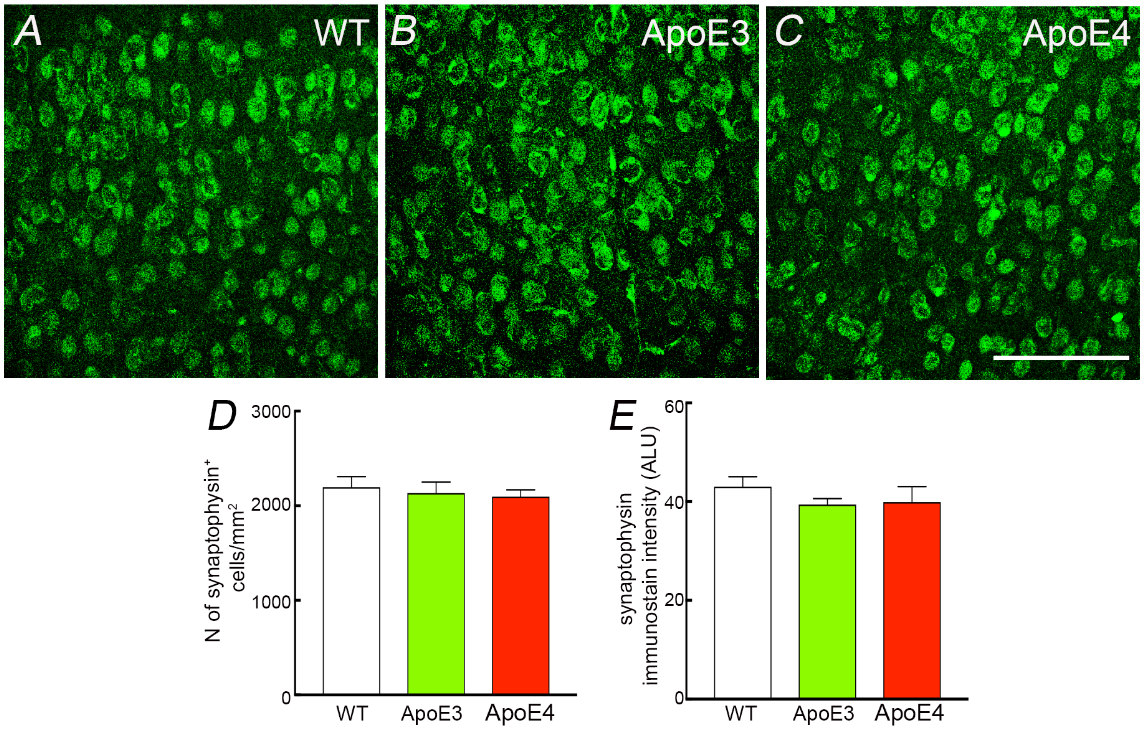

Supplementary Figure 5. The presynaptic marker synaptophysin is not reduced in the somatosensory cortex of ApoE4-TR mice. ***A-C***. Representative images of synaptophysin immunoreactivity in the somatosensory cortex of WT (***A***), ApoE3-TR (***B***) and ApoE4-TR mice (***C***). ***D-E***. Number of synaptophysin$^+$ cells (***D***) and intensity of synaptophysin immunostain (***E***) do not differ among groups. Scale bar in C=100 µm. Data represent mean±sem. $p>0.05$; N=5/group; ANOVA and Tukey's test; ALU, arbitray luminescence units.

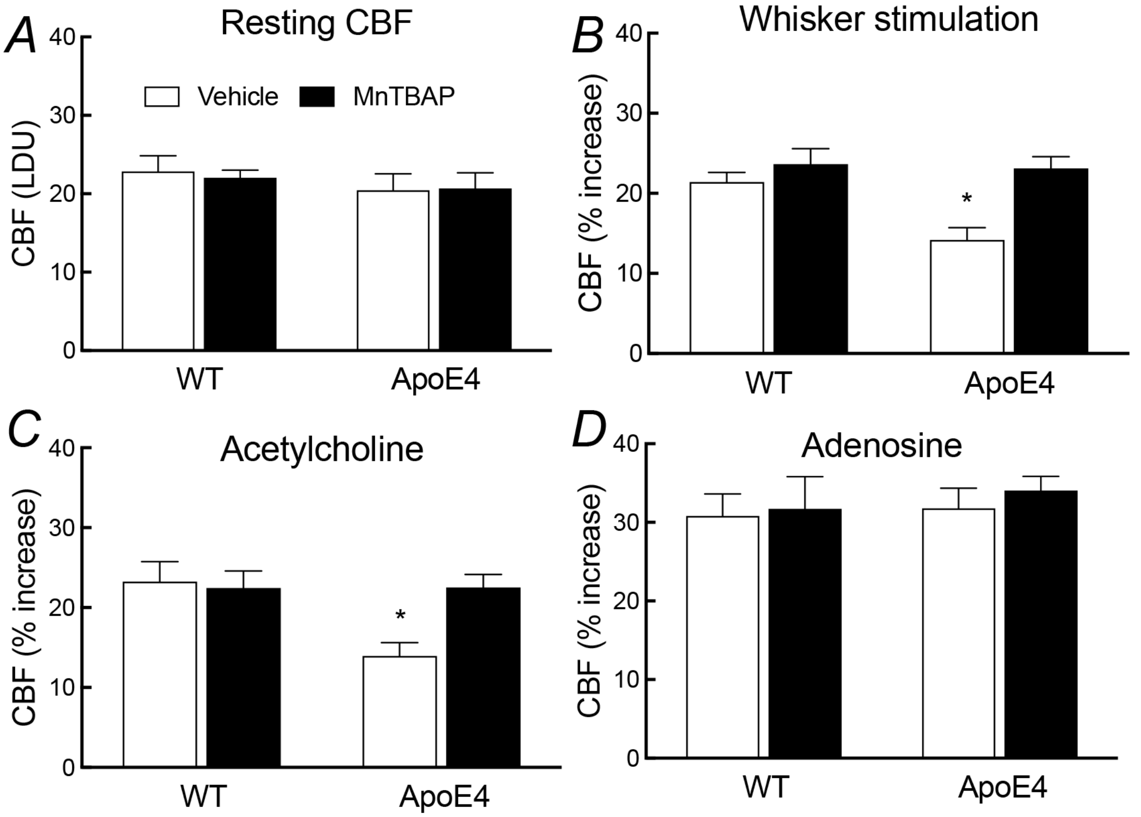

Supplementary Figure 6. The ROS scavenger MnTBAP reverses the cerebrovascular dysfunction in ApoE4-TR mice. Neocortical superfusion with MnTBAP (100μM) does not affect resting CBF (***A***), but reverses the attenuation of the increase in CBF evoked by whisker stimulation (***B***) or neocortical application of acetylcholine (***C***). The CBF response to adenosine (***D***) is not altered. N=5 per group; *P<0.05 from WT and AoE4-MnTBAP; analysis of variance and Tukey's test.

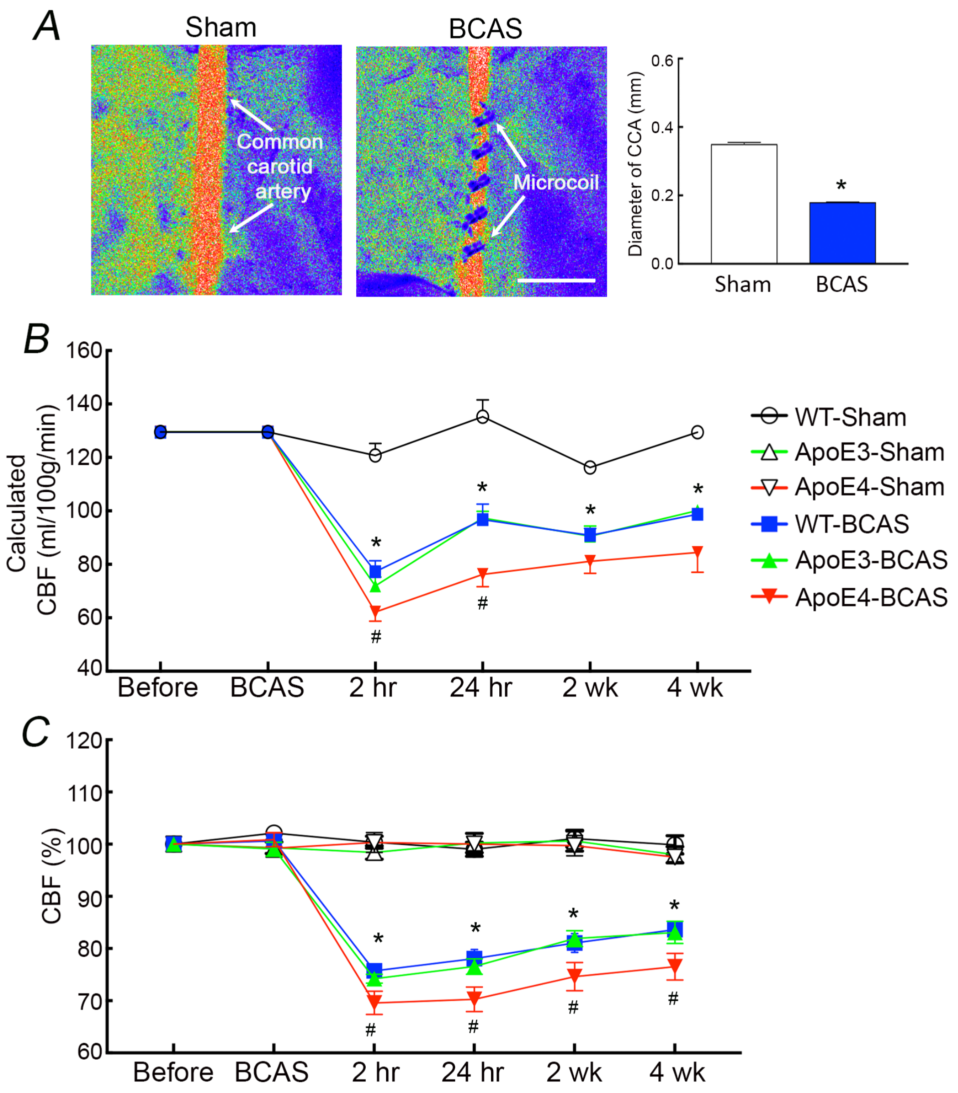

Supplementary Figure 7. Placement of microcoils and time-course of CBF reduction after sham or BCAS in WT, ApoE3-TR, and ApoE4-TR mice. *A*. Laser-speckle image illustrating the reduction in the diameter of the common carotid artery (left panel, arrows) produced by microcoil placement (right panel, arrows); * $p<0.05$ from Sham; N=5/group; t-test. Scale bar=1 mm. ***B***. Time-course of the reduction in neocortical CBF after BCAS. Absolute CBF (ml/100g/min) was calculated from resting CBF values obtained with ASL-MRI (fig. 1***A***) and the % reduction observed with LDF (fig. 3***A***). ***C:*** Time-course of the reduction in relative CBF induced by BCAS, as monitored by laser-speckle imaging (fig. 3B). Right and left CBF values were not different ($p>0.05$) and were combined. BCAS induced a more profound reduction in neocortical CBF in ApoE4-TR than in WT and ApoE3-TR mice. * $p<0.05$ from Sham group; # $p<0.05$ from WT-BCAS and ApoE3-BCAS; N=5/group; one-way ANOVA and Tukey's test.

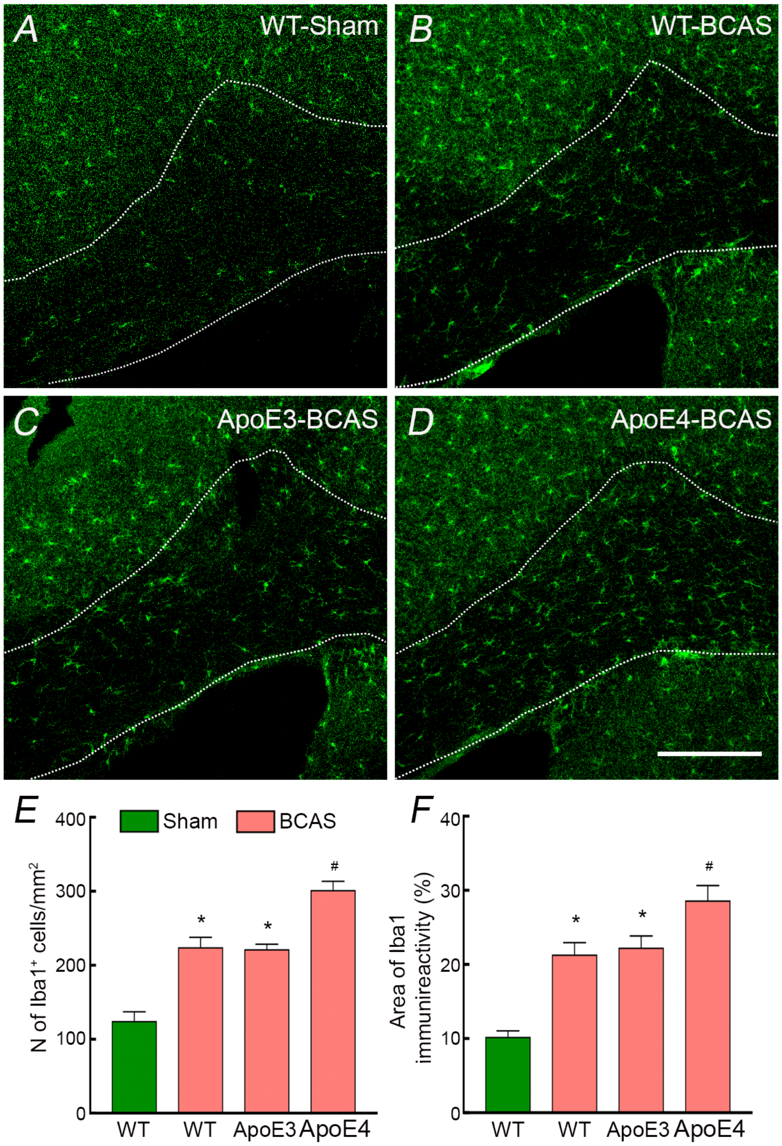

Supplementary Figure 8. ApoE4-TR mice show greater increase in the macrophage/microglia marker Iba1 in corpus callosum after BCAS. ***A-D.*** Representative images of Iba1$^+$ cells in corpus callosum, demarcated by white dotted lines, after sham treatment in WT mice (***A***), and after BCAS in WT (***B***), ApoE3-TR (***C***) and ApoE4-TR mice (***D***). ***E-F***. BCAS increases the number of Iba1$^+$ cells (***E***) and area of the immunostain (***F)*** in all goups, but the effect is more marked in ApoE4-TR mice. Data represent mean±sem. Scale bar in D=250 μm. *p<0.05 from WT-Sham; $^\#$p<0.05 from WT, WT-BCAS and ApoE3-BCAS; N=5/group; one way ANOVA and Tukey test.

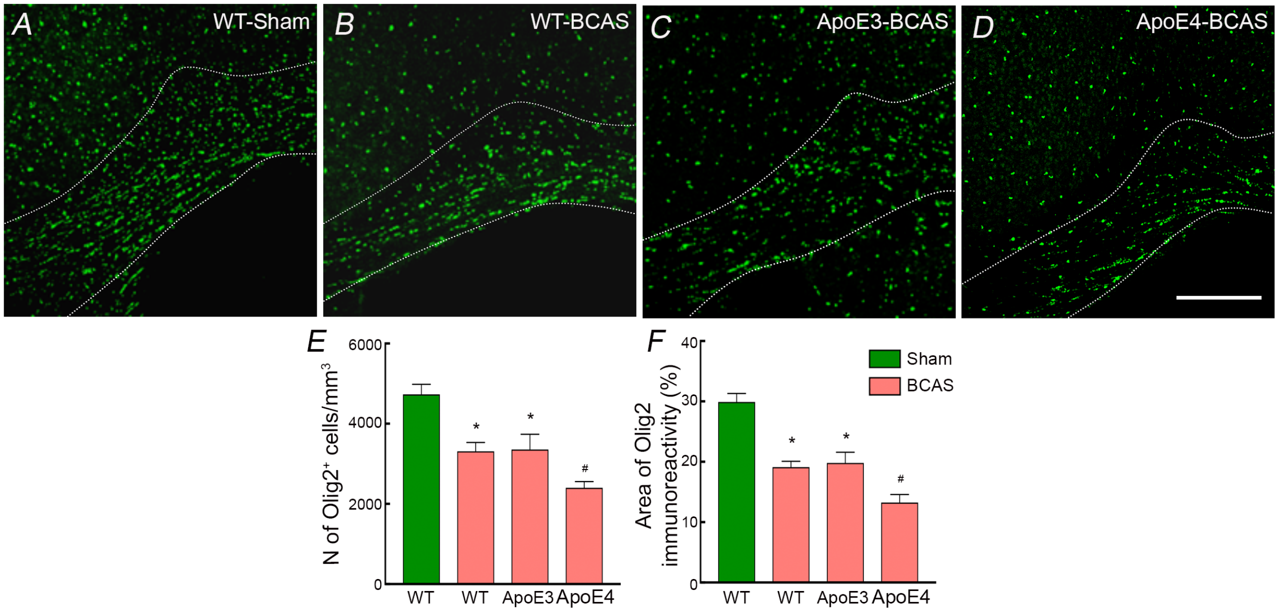

Supplementary Figure 9. ApoE4-TR mice show greater reduction in the oligodendrocyte marker Olig2 in corpus callosum after BCAS. ***A-D***. Representative images of Olig2⁺ cells in corpus callosum, demarcated by white dotted lines, after sham treatment in WT mice (A) and after BCAS in WT (***B***), ApoE3-TR (***C***) and ApoE4-TR mice (***D***). ***E-F.*** BCAS reduces the number of Olig2⁺ cells (***E***) and area of the immunostain (***F***) in all goups, but the effect is more marked in ApoE4-TR. Data represent mean±sem. Scale bar in D=250 μm. *p<0.05 from WT-Sham; #p<0.05 from WT-BCAS and ApoE3-BCAS; N=5/group; one way ANOVA and Tukey test.

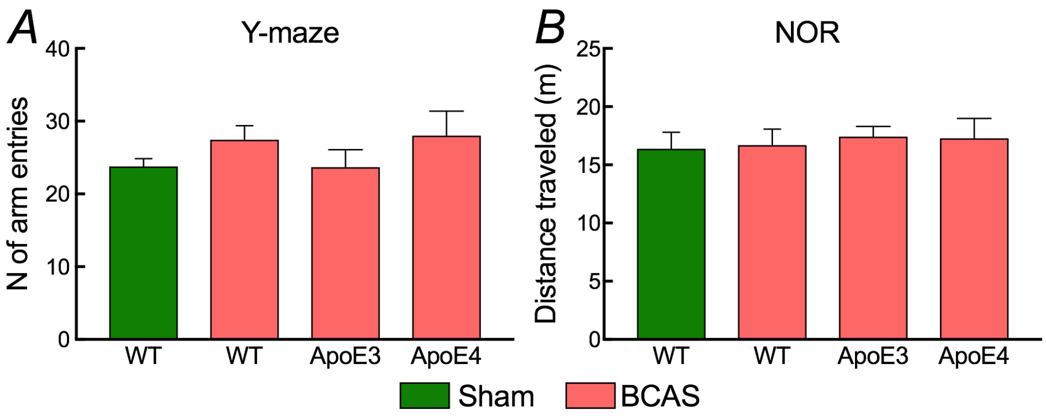

Supplementary Figure 10. Locomotor activity after BCAS or sham surgery. Indices of locomotor activity, recorded during execution of the Y-maze (number of arm entries) (***A***) or novel object recognition test (distance traveled) (***B***), do not differ anong groups after sham or BCAS. $p>0.05$; N=5/group; one-way ANOVA and Tukey's test.

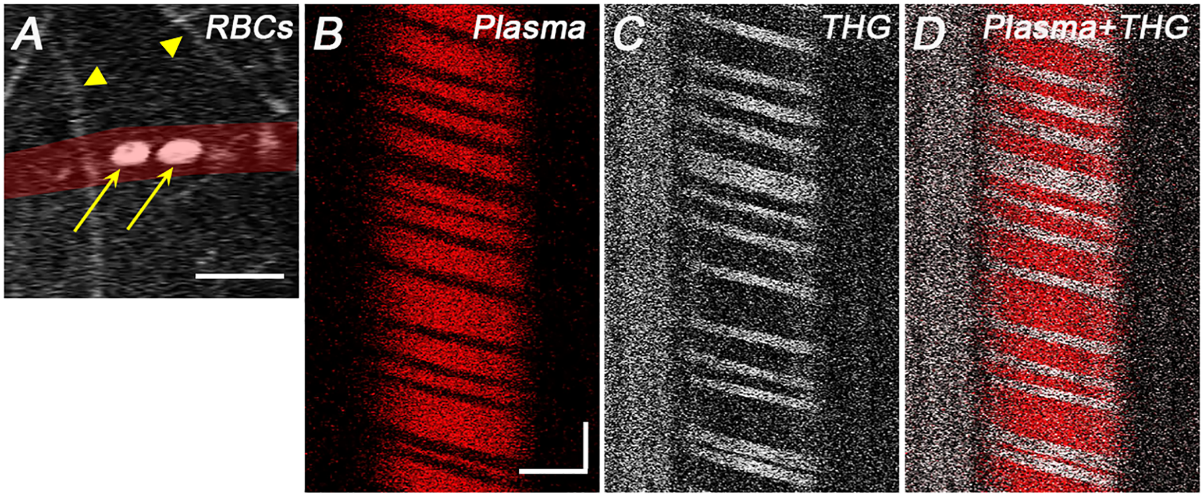

Supplementary Figure 11. Third harmonic generation line-scanning used for red blood cell velocity measurements. ***A***. Imaging of red blood cells (RBCs) (arrows) using third harmonic generation (THG) in a brain capillary (shaded in red). THG is a nonlinear optical process that is enhanced at interfaces between optically dissimilar materials, such as at the interface between blood plasma and RBCs [1]. Note that myelinated axons also produce strong THG [2] and are seen in the image (arrowheads). Images in *B-D* are taken from Figure 4E. Scale bar=10 µm. ***B***. Three-photon excited fluorescence (3PEF) line-scan along the centerline of a cortical blood vessel where FITC-labeled dextran has been intravenously injected to label the blood plasma. The black stripes represent flowing RBCs, which do not take up the dextran dye. Horizontal and vertical scale bars are 10 µm and 50 ms, respectively. ***C.*** Line-scan obtained with THG taken together with the 3PEF data in B. The white stripes, which overlap with the black stripes in ***B***, represent RBCs. The slope of the stripes is proportional to the inverse of the speed. We used a automated algorithm [3] to determine the slope of the streaks and computed an average speed across about 60 s of data. ***D.*** Merged image of ***B*** and ***C*** showing alternating, mutually exclusive red/white stripes representing 3PEF from plasma (red) and THG from RBCs (white).

Supplementary Table 1. List of primary and secondary antibodies

Primary antibodies	Concentration	Catalog N.	Company
**Blood vessel markers**			
Rat anti-mouse cluster of differentiation 31 (CD31)	1:30	553370	BD Pharmingen
Rabbit anti-glucose transporter (Glut1)	1:500	400060	Millipore Sigma
**Axonal, nodal and paranodal markers**			
Mouse anti-contactin-associated protein 1 (Caspr)	1:300	MABN69	Millipore Sigma
Rabbit anti-voltage-gated sodium chaneel (Nav)1.6	1:200	ASC-009	Alomone Labs
Rat anti-myelin basic protein (MBP)	1:500	MAB386	Millipore Sigma
Mouse anti-neurofilament marker SMI312	1:500	837904	BioLegend
Mouse anti-myelin-associated glycoprotein (MAG)	1:500	MAB1567	Millipore Sigma
**Pericyte marker**			
Goat anti-mouse aminopeptidase N/CD13	1:100	AF2335	R&D Systems
**Macrophage/microglial marker**			
Rabbit anti-Ionized calcium binding adaptor molecule 1(Iba1)	1:500	019-19741	Wako
**Neuronal presynaptic marker**			
Mouse anti-human synaptophysin	1:100	MAB5555	R&D Systems
**Oligodendrocyte precursor marker**			
Rabbit anti-oligodendrocyte transcription factor 2 (Olig2)	1:200	AB9610	Millipore Sigma

Secondary antibodies	Concentration	Catalog N.	Company
Donkey Anti-rat IgG (H+L) fluorescein (FITC)	1:200	712-095-153	Jackson ImmunoResearch
Donkey Anti-rabbit IgG (H+L) FITC	1:200	711-095-152	
Donkey Anti-mouse IgG (H+L) Alexa 647	1:200	715-605-151	
Donkey Anti-mouse IgG (H+L) FITC	1:200	715-095-150	
Donkey anti-goat IgG (H+L) FITC	1:200	705-095-147	

Supplementary References

1. Dietzel S*, et al.* Label-free determination of hemodynamic parameters in the microcirculaton with third harmonic generation microscopy. *PLoS One* **9**, e99615 (2014).

2. Farrar MJ, Wise FW, Fetcho JR, Schaffer CB. In vivo imaging of myelin in the vertebrate central nervous system using third harmonic generation microscopy. *Biophys J* **100**, 1362-1371 (2011).

3. Santisakultarm TP*, et al.* In vivo two-photon excited fluorescence microscopy reveals cardiac- and respiration-dependent pulsatile blood flow in cortical blood vessels in mice. *Am J Physiol Heart Circ Physiol* **302**, H1367-1377 (2012).
